# An evaluation of three-dimensional photogrammetric and morphometric techniques for estimating volume and mass in Weddell seals *Leptonychotes weddellii*

**DOI:** 10.1371/journal.pone.0189865

**Published:** 2018-01-10

**Authors:** Roxanne S. Beltran, Brandi Ruscher-Hill, Amy L. Kirkham, Jennifer M. Burns

**Affiliations:** 1 Department of Biology and Wildlife, University of Alaska Fairbanks, Fairbanks, Alaska, United States of America; 2 Department of Biological Sciences, University of Alaska Anchorage, Anchorage, Alaska, United States of America; 3 Department of Ecology and Evolutionary Biology, University of California, Santa Cruz, California, United States of America; 4 College of Fisheries and Ocean Sciences, University of Alaska Fairbanks, Juneau, Alaska, United States of America; Centre National de la Recherche Scientifique, FRANCE

## Abstract

Body mass dynamics of animals can indicate critical associations between extrinsic factors and population vital rates. Photogrammetry can be used to estimate mass of individuals in species whose life histories make it logistically difficult to obtain direct body mass measurements. Such studies typically use equations to relate volume estimates from photogrammetry to mass; however, most fail to identify the sources of error between the estimated and actual mass. Our objective was to identify the sources of error that prevent photogrammetric mass estimation from directly predicting actual mass, and develop a methodology to correct this issue. To do this, we obtained mass, body measurements, and scaled photos for 56 sedated Weddell seals (*Leptonychotes weddellii*). After creating a three-dimensional silhouette in the image processing program PhotoModeler Pro, we used horizontal scale bars to define the ground plane, then removed the below-ground portion of the animal’s estimated silhouette. We then re-calculated body volume and applied an expected density to estimate animal mass. We compared the body mass estimates derived from this silhouette slice method with estimates derived from two other published methodologies: body mass calculated using photogrammetry coupled with a species-specific correction factor, and estimates using elliptical cones and measured tissue densities. The estimated mass values (mean ± standard deviation 345±71 kg for correction equation, 346±75 kg for silhouette slice, 343±76 kg for cones) were not statistically distinguishable from each other or from actual mass (346±73 kg) (ANOVA with Tukey HSD post-hoc, *p*>0.05 for all pairwise comparisons). We conclude that volume overestimates from photogrammetry are likely due to the inability of photo modeling software to properly render the ventral surface of the animal where it contacts the ground. Due to logistical differences between the “correction equation”, “silhouette slicing”, and “cones” approaches, researchers may find one technique more useful for certain study programs. In combination or exclusively, these three-dimensional mass estimation techniques have great utility in field studies with repeated measures sampling designs or where logistic constraints preclude weighing animals.

## Introduction

Body mass dynamics in animals can elucidate critical associations between environmental factors and prey consumption, as mass changes reflect disparities between energy acquisition and expenditure [1, 2]. Seasonal mass fluctuations also provide a crucial metric against which to judge ecological shifts such as intra- and inter-annual prey availability [3]. In species that are logistically complicated to study, accurate mass or volume measurements can be used to help predict how body condition affects other physiological, behavioral, or life-history traits, including thermal balance [4], social dominance [5], mating success [6], fecundity [7], sexual selection [8], and life history evolution [9]. Obtaining mass measurements of marine mammals has enabled researchers to better understand the spatial and temporal dynamics of ocean ecosystems, which are notoriously difficult to sample. For instance, mass measurements have revealed the effects of El Niño conditions on the quality of maternal care in Northern elephant seals *Mirounga angustirostris* [10], density-dependence in New Zealand fur seals *Arctocephalus forsteri* [11], and the sensitivity of pregnancies to maternal energy balance in species with different life history strategies (*A*. *forsteri*, crabeater seals *Lobodon caranophagus*, and grey seals *Halichoerus grypus*) [12]. Links between extrinsic factors and physiologically meditated population dynamics [13] have provided insight into how environmental changes are likely to influence physiological condition [14], maternal attendance [10], and foraging success [15]. For many species, accurate field estimations of mass and volume are key to understanding an animal's condition, physiology, and behavior at the individual and population level.

For many marine mammals, large body sizes [16, 17] and aquatic life histories [18] make it impossible to directly measure body mass or volume using conventional methods. Mass measurements in some species require time-consuming and/or disruptive methods, such as physical and chemical immobilization [1] or luring an animal over a platform scale [19], that are expensive and limit sample sizes. Consequently, marine mammal researchers have been fine-tuning non-invasive mass estimation methods since Usher and Church [20], who initially estimated masses of ringed seals *Pusa hispida* from body lengths and girths. Gales and Burton [21] subsequently developed a method that allowed a seal’s weight and condition to be approximated using morphometric measurements (length, girth, and the thickness of the blubber layer as determined by ultrasound). These measurements allow the seal to be modeled as a series of contiguous truncated cones with a lean core and an outer blubber layer. Masses of the lean and blubber compartments may then be estimated based on calculated cone volumes and expected tissue densities. This truncated cones method has been widely used to predict mass and lipid stores in a range of marine mammal species [1, 22, 23]. Recent modifications to this method that account for elliptical shape (body cross-section) and separately estimate skin and blubber volume have further increased the method’s accuracy in predicting both mass and percent blubber (condition) in free-ranging pinnipeds [24, 25]. Truncated cones and related methods that produce estimates of both core tissue and blubber layer volumes can account for differences in the density of specific tissue stores [25, 26]. Yet estimating body mass from morphometrics does require some animal handling, and in some circumstances less invasive methods may be preferable.

Photogrammetry provides a promising alternative to direct morphometric measurements because it does not require any animal handling, thus limiting disturbance, reducing risk, and allowing for larger sample sizes and more frequent mass estimates for individual animals [27]. Many early photogrammetry studies required the use of custom equipment [19, 28] that limited utility; however, advancements in camera and software technologies have allowed photogrammetric mass estimates in many pinnipeds and other mammal, bird and fish species [14, 29–31]. Waite et al. [27] developed a method to produce a 3D wireframe model from which volume could be estimated, but the technique required multiple, time-synchronous photographs of a still animal, and so was not highly workable in a field setting. More recently, de Bruyn et al. [32] used commercial digital image processing software (PhotoModeler) to create scaled 3-dimensional wireframes of animals from sequential photographs based on substrate reference points. In this method, volume was determined from wire-frame models and mass was estimated using species-specific correction factors determined from the difference between actual weight and photogrammetric mass estimates (e.g., Postma et al. [33]). Only after these correction factors are determined can mass be estimated accurately for animals that are not handled [32, 33].

In all cases, for photogrammetric methods to accurately estimate mass from volume, both the volume and density of the animal must be known with sufficient accuracy; however, obtaining true measures of full-body density or volume is almost always impossible. Correction factors determined from actual mass and photogrammetrically estimated volume can be used to adjust for errors in both of these values, but do not distinquish between the sources of error, nor point to the underlying cause. Unfortunately, to date, studies that use corrective equations typically fail to separate errors due to photogrammetric volume estimates from those associated with estimates of animal density. Therefore, our objective was to evaluate a suite of methods commonly used to estimate mass in marine mammals and to discuss their relative strengths and weaknesses for use under field conditions. To do so, we compared mass, volume, and density estimates from three different methods: 1) Morphometric measurements (“cones”) [24, 25]; 2) 3D photogrammetric analysis corrected for mass overestimation in PhotoModeler via calibration with known mass (“correction equation”) [32]; and 3) a new method we introduce here that modifies the 3D photogrammetric approach by removing a potentially large source of volume overestimation (“silhouette slice”) and then uses a density estimate calculated from actual mass and measured volume.

## Materials and methods

### Ethics statement

Animal handling protocols were approved by the University of Alaska Anchorage and Fairbanks Institutional Animal Care and Use Committee approvals #419971 and #854089. Research and sample import to the United States was authorized under National Marine Fisheries Service Marine Mammal permit #17411. Research activities were approved through Antarctic Conservation Act permit #2014–003.

### Field methods

We obtained conventional mass measurements, morphometric measurements, and photographs with scale bars for 56 adult, female Weddell seals (*Leptonychotes weddellii*) in Erebus Bay, Antarctica (77°S, 165°W). We anesthetized free-ranging animals between November and February 2013–2015 as part of a concurrent study using protocols outlined in Shero et al. [24]. We measured the body mass of each seal by enclosing the animal in a sling suspended from a tripod and electronic scale (MSI-7300 Dyna-Link 2, ±0.25 kg). This measurement was used as the true mass value, against which mass estimates were compared and calibrated.

### Cones method

Animals’ masses were estimated from direct morphometric measurements using the elliptical truncated cones method developed by Shero et al. [24] and modified by Schwarz et al. [25]. Briefly, cumulative curvilinear length, body width and height were measured to the nearest centimeter at eight body sites (ears, neck, axial, sternum, mid, umbilicus, pelvis, ankles). Total curvilinear length was also measured to the nearest centimeter. Dorsal and lateral blubber depths were measured to the nearest 0.01cm with a Sonosite Edge ultrasound and C60x/5-2 MHz convex transducer (SonoSite Inc., Bothell, Washington, USA) at six of the eight body sites, excluding the ears and ankles. Weddell seal skin thickness, skin density, and blubber density were determined using tissue samples salvaged from two freshly (< 48h) deceased adult female Weddell seals found dead of unknown causes in December 2014 and October 2011. Both animals were in normal body condition. Skin samples were collected from 9 sites across the body of the first seal and fixed in formalin. After placing a scale bar perpendicular to the skin surface, we took scaled photographs of these skin samples. We measured skin thickness, the distance from the epidermal surface to the dermis-blubber interface, to the nearest 0.01mm in ImageJ (version 1.49v) and averaged skin thickness values to determine the body-wide mean. We measured skin density and blubber density using a previously-frozen (-80°C) sculp (blubber with skin and hair) sample taken from the lateral flank of the second seal. We extracted and weighed five pieces each of skin and blubber to the nearest 0.001 g, and measured the volume of each to the nearest 0.1 mL using displacement methods [34]. We multiplied total volumes of the blubber and skin compartments of each seal by *MeasuredDensity* values from this study, and core volumes by 1.1 g cm^-3^ [35]. Then, we summed blubber, skin, and core masses for each animal to generate whole body mass estimates.

### Correction equation method

**Fig 1:**
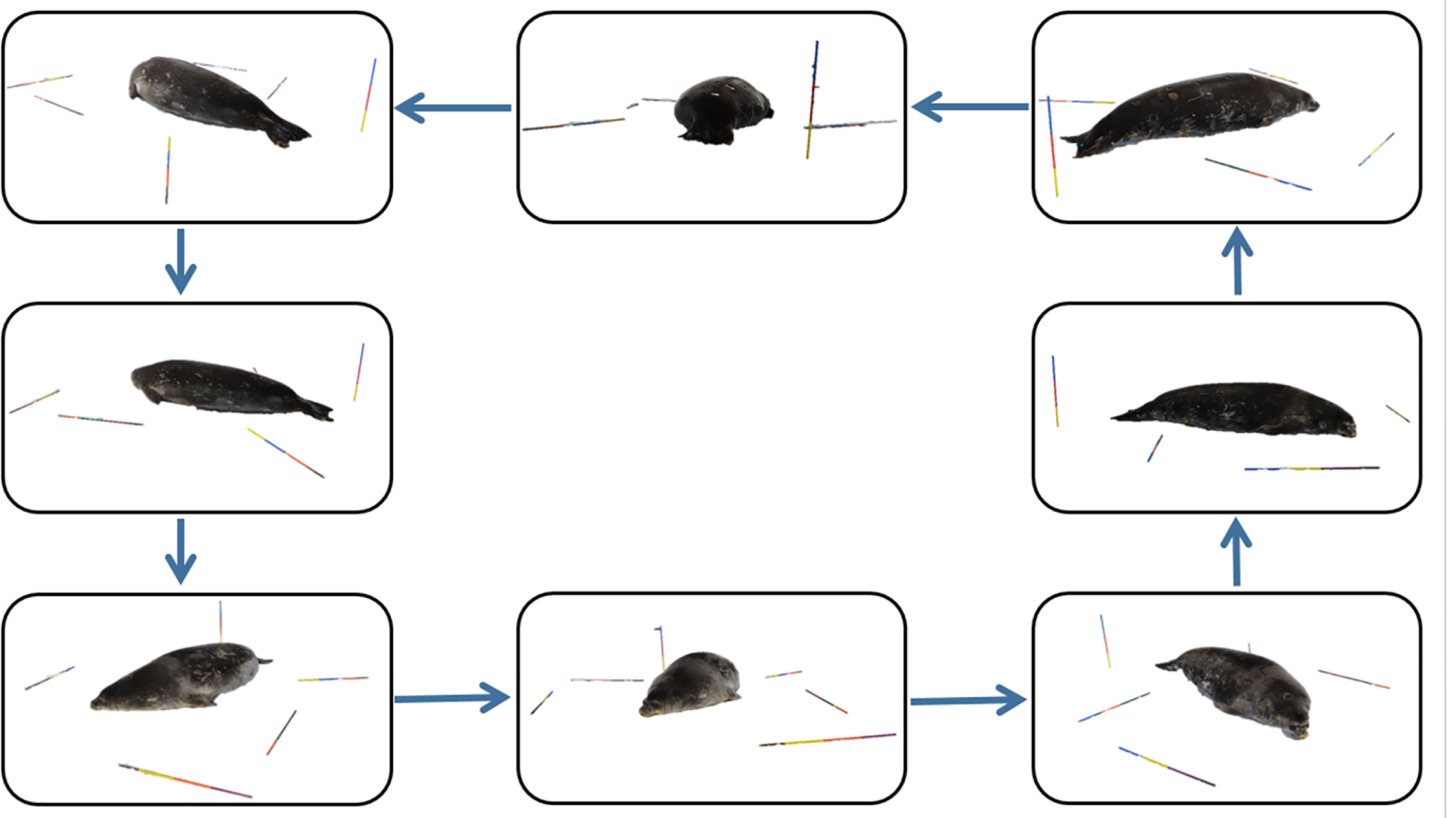
The photogrammetry procedure requires the photographer to slowly circle the seal, taking 8–12 photographs from all possible perspectives (i.e. kneeling, standing, portrait photographs, and landscape photographs). Photos are imported into PhotoModeler and a 3D shape is created by referencing scaled photographs to one another.

We also estimated body volume using a photogrammetric technique. Prior to field work, we calibrated a digital camera (Canon EOS Rebel T3i, 18-55mm lens) using a single- or multi-sheet calibration method (details in de Bruyn et al. [32]). We took images at minimum zoom (18 mm) with auto-rotate and image stabilizer functions disabled to ensure repeatability. When animals were sedated and lying on their ventral surface, we placed six one-meter long rebar rods marked with 25-cm color increments on the ice surrounding the animal to provide a reference point for photogrammetric analysis. These rods replaced the substrate markers used in de Bruyn et al. [32]. We placed one rod vertically and the remaining rods horizontally on the ground circling the seal. The photographer slowly circled the animal taking 8 to 12 photographs from all possible perspectives (e.g., kneeling, standing, portrait photographs, and landscape photographs) (Fig 1). We then used PhotoModeler Pro (Version 2013.0.3, EOS Systems Inc.), Autodesk Meshmixer (10.2.32) and Blender (2.70) to process the photographs. For each seal, we imported photographs as a unique PhotoModeler project associated with the calibrated camera. On each photograph, we marked reference points at each colored scale bar increment. We also outlined the seal silhouette in each photograph excluding the fore flippers and including the rear flippers to ensure consistency across animals, as in de Bruyn et al. [32]. We referenced each scale bar point and seal silhouette to itself across all photographs. Then, we processed the project to orient the camera positions, and set the scale bar to 0.25 meters for one color increment. Finally, we processed the project and measured the volume of each three-dimensional seal silhouette.

We developed a species-specific density estimate to convert photogrammetry-estimated volume to mass. For this “correction equation” method, we calculated an apparent density (*ApparentDensityUnsliced;* g cm^-3^*)* for each individual using Eq 1: $ApparentDensityUnsliced=\frac{{Mass}_{actual}}{VolumeUnsliced}*0.001$ where *Mass*_*actual*_ is the actual mass in kg and *VolumeUnsliced* is the animal volume in m^3^ from PhotoModeler. The *ApparentDensityUnsliced* values for all animals were averaged to produce a species-typical mean density value *MeanDensityUnsliced* (g cm^-3^). This value was then used to derive mass estimates (*MassEstimateUnsliced)* for each animal from their measured volume using Eq 2: *MassEstimateUnsliced* = *VolumeUnsliced* × *MeanDensityUnsliced*. This is an algebraic simplification of the equations used by de Bruyn et al. [32], who calculated an average “correction factor” based on apparent density (true mass/estimated volume) and an ‘assumed density’ of 1.01 that had no effect on their calculation of estimated mass.

### Silhouette slice method

**Fig 2:**
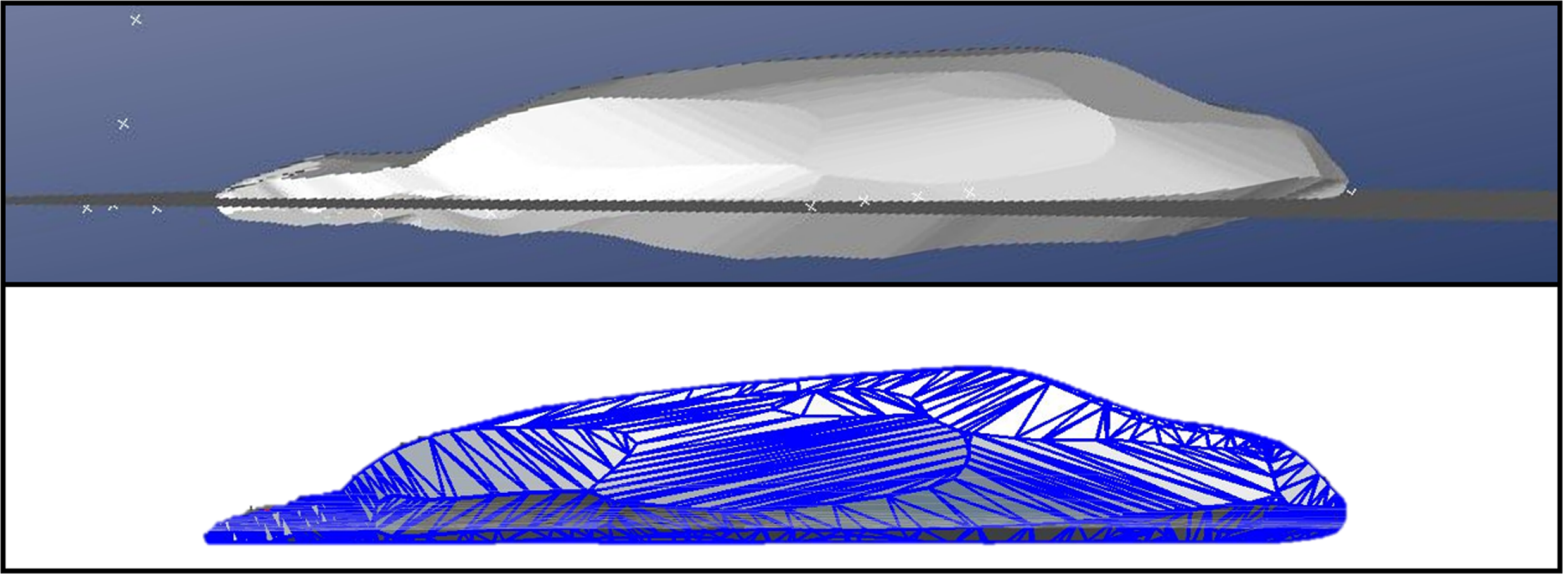
For the “silhouette slice” method, the portion of the seal below the ground plane is identified in PhotoModeler (top panel) and is removed to reduce error (bottom panel).

For the “silhouette slice” method, we further processed the 3D silhouette produced by the program to account for difficulties that PhotoModeler appeared to have in accurately rendering the ventral surface of the seal. This likely occurs because the program cannot cross-reference a photo taken from directly adjacent to the ground, as the program is unable to see all the substrate markers. To differentiate between portions of the modeled seal that were above and below the ground surface, we created a plane in PhotoModeler by selecting all the scale bar points and creating a best fit plane (Fig 2). The new 3D seal shape, including the ground plane, was imported into Autodesk Meshmixer and Blender to split the 3-dimensional seal at the ground plane, slicing off the below-ground volume so that we measured only the above-ground volume. This resulted in a new volume estimate, *VolumeSliced*. Then, we calculated an apparent density (*ApparentDensitySliced;* g cm^-3^*)* for each individual using Eq 3: $ApparentDensitySliced=\frac{{Mass}_{actual}}{VolumeSliced}*0.001.$ The *MeanDensitySliced* (g cm^-3^) was then calculated and used to estimate mass (*MassEstimateSliced)* for each animal using Eq 4: *MassEstimateSliced* = *VolumeSliced* × *MeanDensitySliced*.

### Statistical methods

Prior to analysis, we visually assessed data for outliers and used a Shapiro-Wilk test to assess normality. We used an analysis of variance (hereafter, ANOVA) and TukeyHSD post-hoc test to assess whether each mass estimation method was significantly different from actual body mass. Similarly, we used ANOVAs to compare the volume estimates and apparent densities across methods. We then normalized estimation error of each method by calculating percent error: $Error=\frac{M_{estimate}-M_{actual}}{M_{actual}}*100$ and used an ANOVA to compare the error rates of each method in terms of percent error and kilograms. Additionally, a simple linear regression was performed to regress actual mass against estimated mass to inspect residuals and ensure the variance was homoscedastic. All analyses were conducted in *R* (version 3.2.0) and significance was assessed at α = 0.05. Data are presented as mean ± standard deviation around the mean values (n = 56) for each photogrammetric estimation method.

## Results

### Actual mass

The scale-measured seal masses ranged from 225 to 527 kg (mean ± standard deviation 346 ± 73 kg) (Table 1).

**Table 1:** Comparison of actual mass, parameter estimations, and percent error across estimation methods given as mean ± standard deviation for 56 animals. Mass was estimated using three methods: elliptical “cones”, “correction equation”, and “silhouette slice”. Percent error was calculated as 100*the difference between the estimated mass and the actual mass divided by the actual mass. Superscript letters denote a significant difference in parameters across estimation methods. The sedation, equipment, and time requirements for all methods are noted.

Parameter	Elliptical Cones	Photogrammetry, Correction Equation	Photogrammetry, Silhouette Slice
**Actual Mass (kg)**	346 ± 73	346 ± 73	346 ± 73
**Estimated Volume (m**^**3**^**)**	0.323 ± 0.074 ^a^	0.399 ± 0.082 ^b^	0.346 ± 0.075 ^a^
**Estimated Mass (kg)**	343 ± 76 ^a^	345 ± 71 ^a^	346 ± 75 ^a^
**Apparent Density (g cm**^**-3**^**)**	1.08 ± 0.06 ^¥^ ^a^	0.87 ± 0.05 ^b^	1.00 ± 0.05 ^c^
**Error (kg)**	-3 ± 20 ^a^	-1 ± 20 ^a^	0 ± 19 ^a^
**Error (%)**	1 ± 5 ^a^	0 ± 6 ^a^	0 ± 6 ^a^
**Sedation Required**	Yes	No ^Ω^	No ^Ω^
**Apparent Density Required**	No	Yes	Yes
**Equipment**	Maximum	Moderate	Moderate
**Field Data Collection Time (Minutes)**	30	2–5 ^θ^	2–5 ^θ^
**Data Processing Time (Minutes)**	15	25–40	30–45

^Ω^ Animals were sedated during this study to measure mass for validations and apparent density calculations.

^¥^ To ensure comparability across methods, density for the elliptical cones method was calculated using the estimated volume and actual mass, rather than incorporating the lean and blubber volumes and densities.

^θ^ The time required for field data collection will be slightly more for non-sedated animals (~5 minutes) than it was in this study (~2 minutes).

### Processing time

For each animal, the elliptical cones process (“cones” method) required an estimated 90 minutes of animal handling time (~30 minutes of direct morphometric measurements within a 90-minute anesthesia procedure) and an additional 15 minutes of data processing time (Table 1). The photogrammetric analysis process (“correction equation” method) required approximately 2 minutes in the field and 25–40 minutes on the computer (Table 1). The slicing process (“silhouette slice” method) took approximately five minutes per animal beyond that required to generate the first silhouette in PhotoModeler (approximately 30–45 total minutes per project) (Table 1).

### Volume

Mean overall project residual error for individual projects (n = 56) was 2.650 pixels (range: 0.729 to 4.844). Volume estimates for the “cones” method (0.323 ± 0.074 m^3^, Table 1) ranged from 5 to 9% (7 ± 1%) skin, 11 to 36% (22 ± 1%) blubber, and 57 to 82% (71 ± 5%) core. Skin thickness was 6.94 ± 0.99 mm, skin density was 1.162 ± 0.057 g cm^-3^, and blubber density was 0.920 ± 0.026 g cm^-3^ for the two deceased Weddell seals. The raw photogrammetric volume estimates used for the “correction equation” method (0.254 to 0.600 m^3^) were significantly larger than volume estimates used for “silhouette slice” (0.223 to 0.547 m^3^) and “cones” (0.215 to 0.520 m^3^) methods (ANOVA, df = 167, N = 168, F-value = 14.28; Tukey HSD post-hoc, *p* = 0.001 and *p*<0.0001, respectively), which were not significantly different from each other (ANOVA, Tukey HSD post-hoc, *p* = 0.254). The was a relatively strong positive relationship between percent blubber measured by ultrasound and volume estimates (linear regression, R^2^ = 0.48 for “silhouette slice” volume, R^2^ = 0.43 for “correction” volume, and R^2^ = 0.44 for “cones” volume).

### Apparent (estimated) density

**Fig 3:**
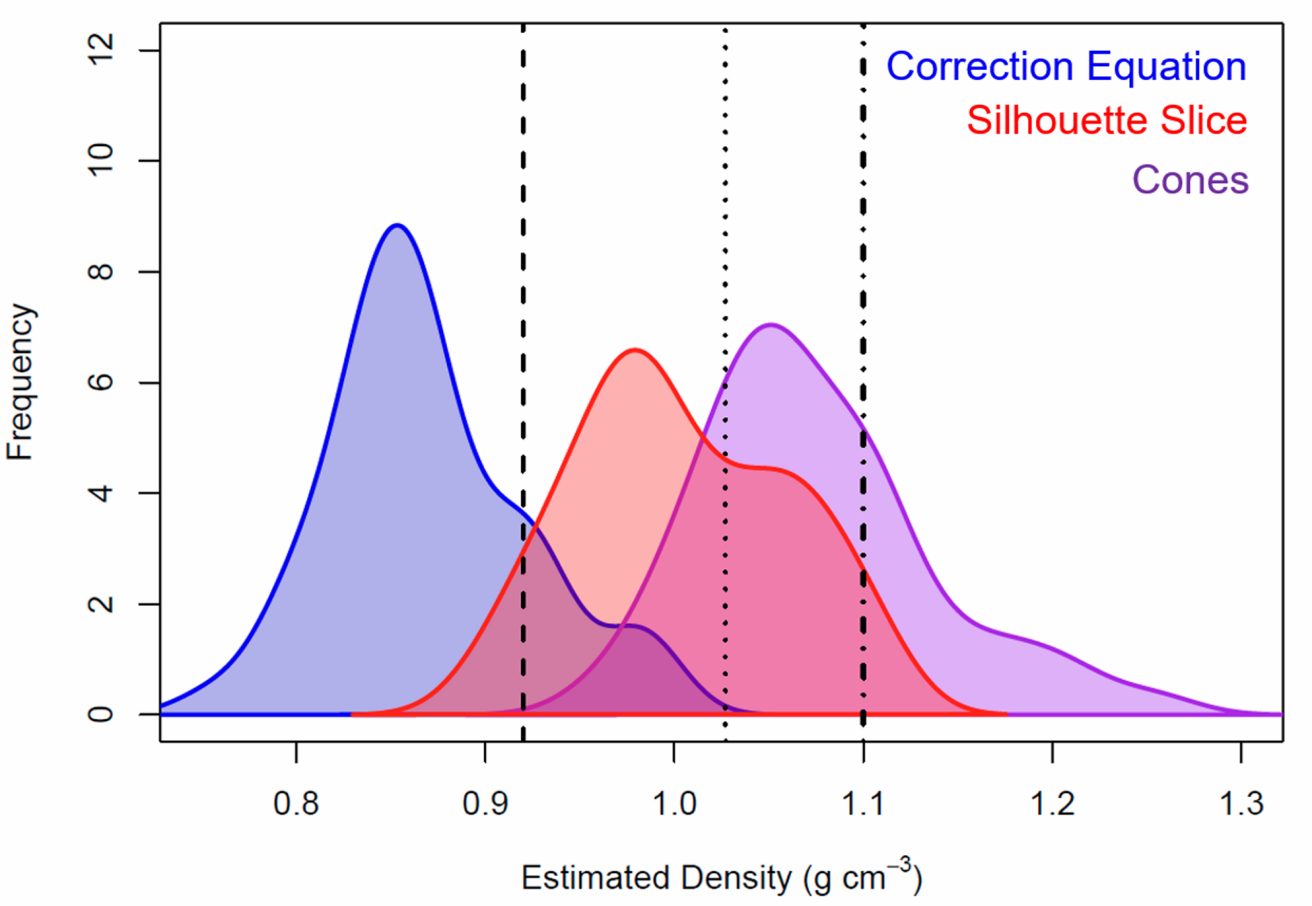
Estimated body density for adult, female Weddell seals calculated from actual body mass and estimated volume. The elliptical “cones” method estimated a higher density than both “correction equation” and “silhouette slice” methods. For reference, vertical lines show the density of seawater (black dotted line, 1.027 g cm^-3^ [36]), blubber (black dashed line, 0.920 g cm^-3^, this paper), and lean tissue (black dashed-dotted line, 1.1 g cm^-3^ [35]).

Combining actual seal masses with volume estimates for the “cones”, “correction equation”, and “silhouette slice” methods led to estimated (apparent) tissue densities of (minimum to maximum) 0.96 to 1.25 g cm^-3^ for *ApparentDensityCones*, 0.76 to 0.99 g cm^-3^ for *ApparentDensityUnsliced*, and 0.90 to 1.11 g cm^-3^ for *ApparentDensitySliced*, respectively (Fig 3). Apparent densities were significantly different across all methods (ANOVA, df = 167, N = 168, F-value = 199.4; Tukey HSD post-hoc, *p*<0.0001 for all). The *MeasuredDensity* value from the “cones” method (derived using volume and actual blubber, skin, and lean densities) was 1.04 to 1.08 g cm^-3^. Surprisingly, there was no significant relationship between an individual’s apparent density and percent blubber (linear regression, R^2^<0.05 for all methods).

### Estimated mass

**Fig 4:**
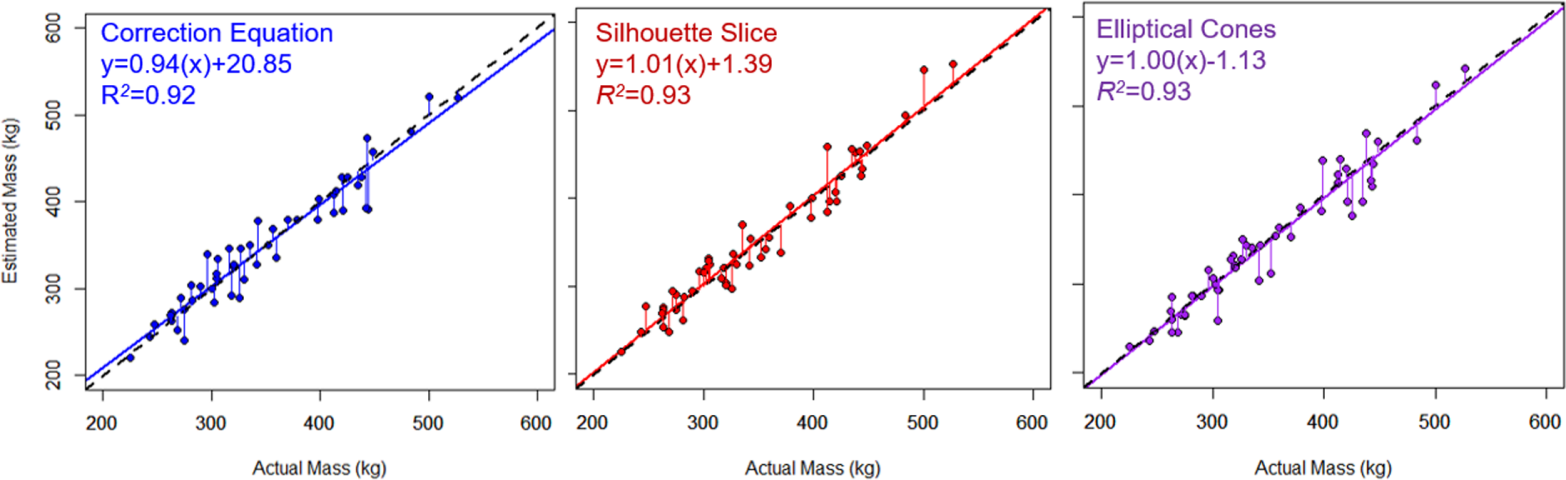
Regressions between actual and estimated mass for the three methods discussed: The equations calculated from de Bruyn et al. [32] (“correction equation”, left panel, *p*<0.0001), the above-ground estimation (“silhouette slice”, middle panel, *p*<0.0001), and the truncated cones method (“elliptical cones”, right panel, *p*<0.0001). Black dashed lines show the 1:1 relationship between estimated and actual mass, whereas colored solid lines show the regression for each method. Horizontal lines show the offsets between data points and the 1:1 line.

**Fig 5:**
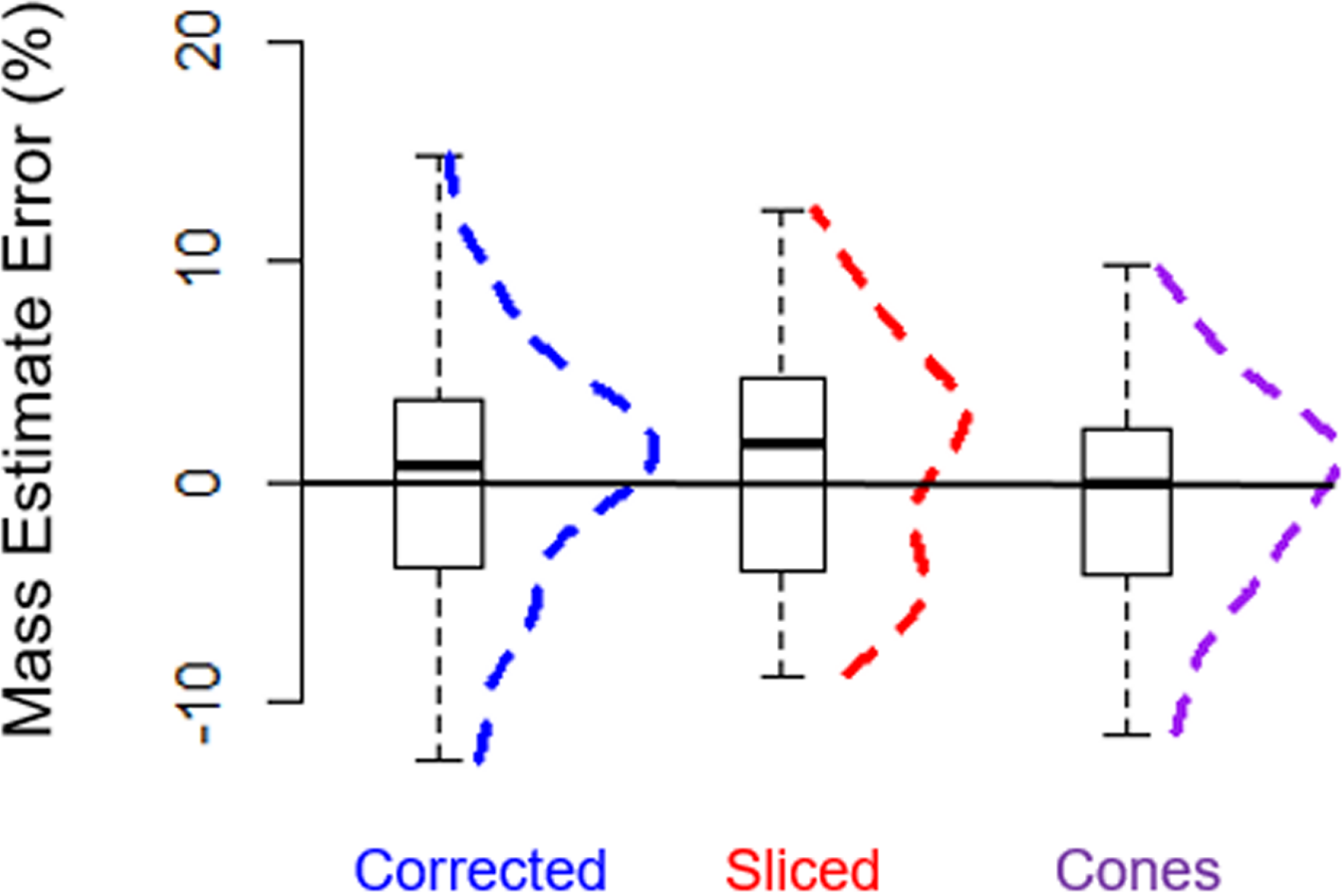
Boxplots of the percentage error for each estimation method with frequency distributions overlaid as dashed lines. Mass estimates from each method were not significantly different than actual mass in the Weddell seals.

The “cones”, “silhouette slice”, and “correction equation” mass estimates were not significantly different from actual mass (ANOVA, Tukey HSD post-hoc, *p* = 0.999; Table 1, Fig 4). All three estimation methods produced mean mass estimate errors of less than 1.0%; however, there was a relatively wide spread, with standard deviations in calculated error of 5, 6, and 6% for each of the three methods, respectively (Fig 5). Root mean square error values for “cones”, “correction equation”, and “silhouette slice” estimations regressed with actual mass were 19.585 kg, 19.062kg, and 19.219 kg, respectively, indicating similar levels of precision.

## Discussion

### Summary

Here, we compare several commonly-used morphometric and photogrammetric methods for three-dimensional volume and mass estimation in pinnipeds. Photogrammetry has long been used to minimize invasiveness and maximize accuracy of mass estimation; however, previous studies have reported a consistent, positive bias in photogrammetric mass estimates (see references in de Bruyn et al. [32]). Directly estimating density using the uncorrected, unsliced photogrammetry allowed us to tease apart the contribution of volume and density to the uncertainty surrounding mass estimates. In the current study, the elliptical “cones”, “silhouette slice”, and equation “correction equation” methods each estimate actual mass with relatively high accuracy, although they achieve this in different ways. Methodologies can introduce error during two stages of mass estimation: during the initial volume estimation, and/or in the density value that is used to calculate mass from volume. Since ‘true’ volume or density values are not available against which to compare estimates, determining the ‘best’ method is not possible; instead, we evaluate the degree to which each method provides reasonable volume and density metrics. Finally, we consider the relative strengths and weaknesses of each approach under different field scenarios (Table 1).

The “cones” method estimated the lowest volume and highest apparent density relative to the other two methods, whereas the “correction equation” method produced the highest volume estimate and lowest apparent density. The “silhouette slice” method produced volume estimates and apparent density that were intermediate to the other two methods. The apparent tissue density (0.87 g cm^-3^; Table 1) required to align estimated volume with directly measured mass in the “correction equation” method was unrealistically low. We can thus conclude that volume was consistently overestimated. We propose that erroneous below-ground volume was the likely reason for the overestimate in photogrammetric volume estimation. A novel “silhouette slice” method for constraining the 3D seal volume to above-ground space improved the ability of the uncorrected photogrammetric method to estimate volume and provided volume estimates comparable to direct morphometric measurements (“cones” method) before the apparent density values were incorporated. Consequently, the silhouette slice approach yielded accurate mass (within 0 ± 5%; see Fig 4) and realistic density estimates. Mass estimates from the other two volume estimates also did not differ significantly from actual mass or from each other, suggesting that they are also viable alternatives to direct mass measurements.

### Apparent density values

The accuracy of the “cones” mass estimation method was likely improved by the incorporation of blubber depths into the density value that is used to convert from volume to mass. Whereas the “correction equation” and “silhouette slice” methods used an average seal body density value to convert volume to mass, the “cones” method involved calculating density of individuals by combining directly measured lipid and lean volumes with tissue-specific density estimates. Calculating individual body density values using the “cones” method can help to reduce uncertainty in body mass estimates across individuals with a range of body compositions [24]. Relative to cones, the “silhouette slice” method produced a slightly lower apparent density (1.00 g cm^-3^).

Although density is nearly impossible to measure in free-ranging animals, realistic density values can be deduced by evaluating physical properties of the marine environment. For instance, researchers have shown that while seal buoyancy fluctuates with body composition, Weddell seals are probably positively buoyant (seal density less than seawater density; 1.027 g cm^-3^ [36, 37]) before lung collapse (in shallower dives), as evidenced by their use of stroke-and-glide swimming during descent and prolonged glide during ascent [38]. Williams et al. [39] found that after a certain depth, Weddell seals may be negatively buoyant as evidenced by gliding locomotion for descent and stoking locomotion for ascent. Based on the assumption that Weddell seals are positively buoyant when lungs are not collapsed (e.g., during haul out periods, as in our study), Weddell seal apparent densities from the “silhouette slice” method are plausible, whereas the “cones” method likely overestimates apparent densities and the “correction equation” likely underestimates apparent densities. Thus, removing the below ground portion (“silhouette slice” method) leads to a more reasonable apparent density value than the “correction equation” method. Note that the methods in this study do not account for the very low densities of brain (~5% body mass [40]) and lung tissues in seals.

### Volume estimates

The uncertainty in body mass estimates contributed by unknown body density can be avoided by using photogrammetric body volume measurements as an independent metric rather than by using them to estimate mass. Indeed, volume is a useful parameter for many research objectives, such as modeling heat flux to the environment (e.g., penguin thermoregulation [41]), understanding the allometry of anatomical features (e.g., volume-specific blood volume [42]), and determining how behavioral processes scale with body size (e.g., whale engulfment capacity [43]). Mass estimates from photogrammetry are derived entirely from volume, with the additional requirement of an apparent tissue density that adds uncertainty. Based on the realistic shape of the 3D silhouettes (Fig 2), we believe that the “silhouette slice” method provided the more believable volume estimates (0.346 ± 0.075 m^3^; Table 1) between the photogrammetric estimation methods we compared. Because there are no true measures of density or volume, we assumed that the realistic silhouette volumes were accurate and thus used actual mass measurements to calculate apparent density values. Due to the erroneous below-ground volume assumed by PhotoModeler, the apparent density needed to convert volume to actual mass is unrealistically low, suggesting that the “correction equation” method overestimated total body volume (0.399 ± 0.082 m^3^; see Table 1). We therefore recommend using the “silhouette slice” method to estimate volume in large mammals that have large areas of contact with the ventral surface. Though it provides a tangible metric, mass estimates should be used with caution when an uncertain apparent density is used to convert volume to mass.

The photogrammetric methods appeared to capture seal body shape more precisely because the silhouette method integrates continuous measurements rather than extrapolating the eight discrete measurements in the morphometric “cones” method. Additionally, the higher “cones” *ApparentDensity* values may be due in part to an underestimate of total body volume in this method (0.323 ± 0.074 m^3^; see Table 1) because it did not account for the front or rear flippers [24]. Despite these differences, there was a linear relationship between percent blubber and volume estimates for all methods, suggesting that the three-dimensional silhouette shapes were capturing the “slumping” of fatter seals. If the volume estimate is accurate, then apparent density should reflect actual animal density, and can be informative within an ecological context. Larger blubber volume would cause more pronounced “slumping” [24, 25, 44] and a relatively larger ventral surface where the animal contacts the ground that would be inaccurately modeled to extend below the ground plane. In this study, we did not detect a relationship between an individual’s apparent density and percent blubber. This may be due to measurement error and/or variation in internal lipid reserves, which are substantial in this species [24].

Volume estimate error can result from any deviation in substrate rugosity [28, 32], animal position [19], or angle of the scale bar relative to the camera [28, 45]. In the current study, the relative flatness and stability of the ice compared to other substrates, such as sand [32], provided an opportunity to take unobstructed photographs. Additionally, the study seals were anesthetized, so all individuals were lying flat and extended with limited or no mobility. Thus, this study system was ideal for comparing existing photogrammetric analysis techniques to our new “silhouette slice” technique; however, we recognize that the immobility of sedated seals in this study may have resulted in lower than expected error estimates. We note that this 3D photogrammetry method has been successfully utilized in Weddell seals by opportunistically taking photographs of sleeping and unresponsive animals (K. Macdonald, J. Rotella, B. Garrott, pers comm). Not all field studies will be as controlled, and natural systems are likely to introduce more error to the photogrammetric results. In situations where unmanned aircraft systems (UAS) can be used and volume estimates are not required, mass estimates may be obtained allometrically rather than using volume and density (D. Krause, pers comm). We note that UAS photogrammetry may be more appropriate for studies of less approachable species (e.g. leopard seals *Hydrurga leptonyx*).

### Field applications of morphometric and photogrammetric methods

The differences in data collection and analytical methodologies between the three techniques are likely to render certain methods more useful for certain study programs. For instance, the most notable methodology difference between this study and de Bruyn et al. [32] was the use of six bars rather than many opportunistic substrate markers. This effectively reduced fieldwork setup effort and analytical processing time. However, because the “silhouette slice” process required scale bars to be placed parallel to the ground surface (Fig 1), the “correction equation” method may be more appropriate for animals on more rugose substrates. Additionally, the uncertainty surrounding the species-specific *ApparentDensity* depends on the number of individuals used to calculate that value. With our data, an average *ApparentDensity* drawn from five animals would have ranged (difference between maximum and minimum *ApparentDensity* divided by the mean *ApparentDensity*) by ~13% whereas the range would be ~3% if drawn from 25 animals. Thus, researchers should take care to weigh a sufficient sample of animals so that *ApparentDensity* values converge around the presumably true mean. If it is not possible to weigh enough animals, using the “cones” method with *MeasuredDensity* values is a promising alternative, if study logistics allow blubber depth measurements to be obtained.

Given that no significant difference was found between actual and estimated mass, any of the mass estimation methods described here can be used in field studies where logistic constraints preclude weighing animals, so long as researchers choose a reasonable apparent density to estimate mass from volume. The advantage of the “silhouette slice” method is transparency, where error mechanisms are clearly defined. Further, if a species-specific apparent density value is known, the “silhouette slice” method should be used as it produced the smallest error across all estimates. Alternatively, the “cones” method had the narrowest error distribution and is a plausible substitute to mass measurements if the morphometric measurements can be obtained because animals are sedated. Finally, by incorporating blubber depth measurements, the “cones” method can be used to directly measure body condition, which is of paramount importance in studies of behavior, ecology, life history, and demography. Using these methods, volume estimates can fill in knowledge gaps of year-round energy dynamics in studies that have repeated measures sampling designs or for animals that are too heavy to weigh (e.g., free-ranging elephants [46]), require intensive and potentially stressful weighing methods (e.g., captive manatees [47]), or are in locations with limited accessibility (e.g., stranded whales [48]).

## Supporting information

S1 Dataset Summarized Weddell seal photogrammetric data. Mass measurements, photogrammetric volume estimates, and density estimates are provided for each individual.
(CSV)
